# A Treatment Option for Symptomatic Chronic Complete Internal Carotid Artery Occlusion: Hybrid Surgery

**DOI:** 10.3389/fnins.2020.00392

**Published:** 2020-04-28

**Authors:** Yunna Yang, Xingju Liu, Rong Wang, Yan Zhang, Dong Zhang, Jizong Zhao

**Affiliations:** ^1^Department of Neurosurgery, Beijing Tiantan Hospital, Capital Medical University, Beijing, China; ^2^China National Clinical Research Center for Neurological Diseases, Beijing Tiantan Hospital, Capital Medical University, Beijing, China; ^3^Center of Stroke, Beijing Institute for Brain Disorders, Capital Medical University, Beijing, China; ^4^Beijing Key Laboratory of Translational Medicine for Cerebrovascular Disease, Beijing Tiantan Hospital, Capital Medical University, Beijing, China; ^5^Department of Neurosurgery, Beijing Chaoyang Hospital, Capital Medical University, Beijing, China; ^6^Savaid Medical School, University of the Chinese Academy of Sciences, Beijing, China

**Keywords:** internal carotid artery occlusion, hybrid surgery, carotid endarterectomy, endovascular intervention, surgical method

## Abstract

**Background:**

Internal carotid artery occlusion (ICAO) is a relatively uncommon but important cause of transient ischemic attack and cerebral infarction. The majority of cases of symptomatic ICAO requires surgical treatment. In this study we performed an investigation of the efficacy and safety of hybrid surgery which is a surgical method for symptomatic chronic complete ICAO.

**Methods:**

Fifty-five patients with symptomatic chronic ICAO treated by hybrid surgery from 2016 to 2019 were included. We recorded and analyzed the patients’ clinical characteristics, angiographic data, recanalization rate, complications, and outcomes. Catheter angiography or computed tomography angiography was used to assess the patency of the recanalized ICA during follow-up.

**Results:**

The total success rate of recanalization was 78.2% (43/55). The occlusions were significantly shorter in the success than failure group (5.40 ± 1.50 vs. 7.56 ± 0.99 cm, respectively; *P* < 0.001). The median duration of ICA occlusion was significantly shorter in the success than failure group (90 vs. 200 days, respectively). The success rates of distal ICA recanalization at the petrous segment or below, cavernous segment, and clinoid segment or above were 100, 33.3, and 14.3%, respectively (*P* < 0.001). Multivariate analysis showed that the level of distal ICA reconstitution was the only factor affecting the recanalization success rate. Periprocedural complications included hyperperfusion syndrome (*n* = 1) and laryngeal nerve injury (*n* = 1). ICA reocclusion occurred in one patient (2.3%). Significant postoperative improvement in symptoms was observed in the success group, with a median modified Rankin scale score of 0 at the 3-month follow-up compared with before recanalization (median, 1) (*P*<0.001).

**Conclusion:**

Hybrid surgery might be safe and effective for patients with symptomatic chronic complete ICAO. The level of distal ICA reconstitution is a predictor of successful recanalization in hybrid operations.

## Introduction

With the aging of the population in China, the incidence rate of chronic internal carotid artery (ICA) occlusion (ICAO) is estimated to be substantial (Ma et al., 2016). ICAO is associated with a 6–14% annual risk of transient ischemic attack (TIA) or stroke, despite medical treatment (Flaherty et al., 2004; Persoon et al., 2011), and a higher risk is present in patients without sufficient collateral compensation (Grubb et al., 1998). The best treatment for symptomatic chronic ICAO is still controversial, surgical treatment could be indicated if pharmacotherapy fails or as prophylaxis therapy in high-risk patients. In earlier studies, extracranial-intracranial (EC-IC) artery bypass, as a low-flow bypass, showed no superiority over medical therapy and didn’t reduce the risk of recurrence ipsilateral ischemic stroke (EC/IC Bypass Study Group, 1985; Powers et al., 2011). Carotid endarterectomy (CEA) and endovascular interventions (EI) are considered to be effective therapeutic approaches to recanalize the occluded ICA, but they have shown low success rates in previous studies (Paty et al., 2003; Lin et al., 2008; Chen et al., 2016). Hybrid surgery in which CEA for a proximal ICAO is combined with EI for a distal ICAO in one session is reportedly a feasible and effective procedure with a higher success rate (Shih et al., 2013; Jiang et al., 2019; Li et al., 2019).

In previous studies, hybrid surgery was performed to treat tandem disease of the carotid bifurcation and arch vessels, and a high success rate of 97% was achieved (Sfyroeras et al., 2011). Hybrid surgery is being increasingly performed for treatment of chronic ICAO. It can remove hard plaques and help endovascular devices to recognize and traverse the true lumen in the distal ICA simultaneously. In the present study, 55 patients with chronic occlusion of the ICA underwent hybrid operation procedures in the same operative session in a hybrid operating room. The patients’ clinical information, radiologic characteristics, complications, and outcomes were analyzed, and factors affecting the success rate were investigated.

## Materials and Methods

### Patient Selection

Consecutive patients with symptomatic chronic ICAO who underwent CEA in conjunction with EI from March 2016 to March 2019 were enrolled in this study. All patients who presented with ipsilateral ischemic symptoms had failed with maximal medical therapy. Medical therapy usually included dual antiplatelet or anticoagulation plus antiplatelet. Patients were diagnosed by Doppler ultrasound, computed tomography (CT) angiography (CTA), magnetic resonance (MR) angiography (MRA), and digital subtraction angiography (DSA). Chronic complete occlusion was defined as a duration of ≥4 weeks between diagnosis and treatment. The specific inclusion criteria were as follows: The patients had recurrent ipsilateral ischemic symptoms despite medical treatment, but had no new ischemic stroke (≥4 weeks); the ipsilateral middle cerebral artery was patent, and no smoke-like blood vessel was present; cerebral hemodynamics was impaired with ‘misery perfusion’ or in stage II hemodynamic failure, including increasing mean transit time (MTT more than 4 s), decreased cerebral blood flow (CBF) ratio (symptomatic side/asymptomatic side <0.95) and increasing oxygen extraction fraction (OEF) ratio (ipsilateral side/contralateral side >1.13) in preoperative perfusion CT (CTP)/MR imaging (MRI) or positron emission tomography (PET) (Powers et al., 2011; Ma et al., 2016); and the proximal occlusion of the ICA had no stump or tamper. The exclusion criteria were as follows: the presence of severe systemic disease that prevented surgery and anesthesia; the presence of heart disease (unstable angina or acute myocardial infarction), bleeding disorder, or a contraindication to aspirin, clopidogrel, heparin, or iodine contrast; and older patients with end-stage disease or in poor neurologic condition. This retrospective study was approved by the institutional review board, and written informed consent was obtained from all participants.

### Preprocedure Management

**FIGURE 1:**
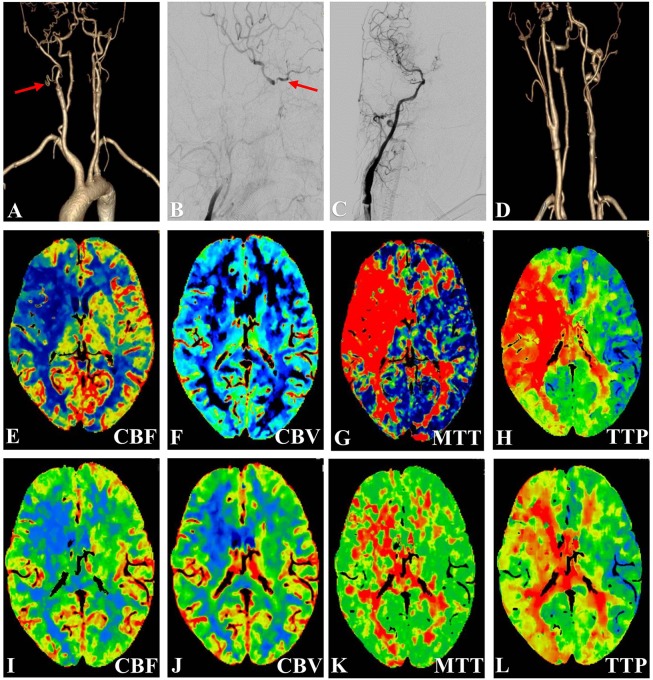
Representative Case 1. A 67-year-old woman with right-sided limb weakness underwent hybrid surgery for internal carotid artery occlusion (ICAO). **(A)** Preoperative computed tomography angiography (CTA) confirmed total occlusion (arrow) of the right ICA without a stump and **(B)** with filling at the ophthalmic segment. **(C)** Digital subtraction angiography (anteroposterior view) showed successful recanalization after deployment of the stent. **(D)** CTA showed a patent ICA at 3 months after the surgery. **(E–L)** Preoperative and postoperative perfusion CT. Postoperative perfusion CT showed improvements in the **(I)** cerebral blood flow (CBF), **(K)** mean transit time (MTT), and **(L)** time to peak (TTP) after recanalization [compared with low CBF in **(E)**, high MTT in **(G)**, and delayed TTP in **(H)**).

**FIGURE 2:**
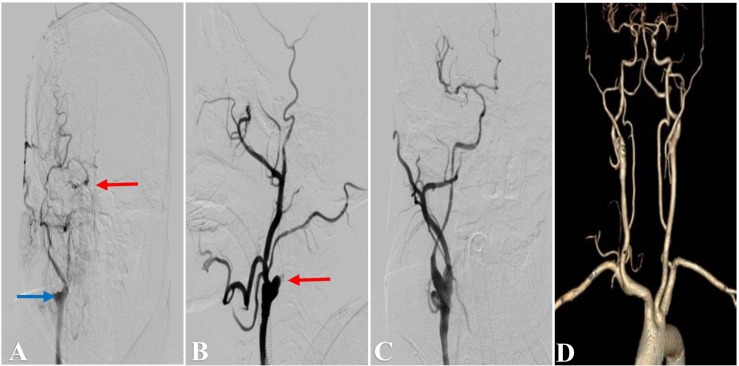
Representative Case 2. A 51-year-old man with left-sided limb weakness underwent hybrid surgery for internal carotid artery occlusion (ICAO). **(A)** Preoperative angiography showed right ICA occlusion (blue arrow) with filling at the cavernous segment (red arrow). Intraoperative angiography showed **(B)** an artificial stump after carotid endarterectomy (red arrow) and **(C)** successful recanalization after deployment of the stent. **(D)** Computed tomography angiography showed a patent ICA at 3 months after the surgery.

**FIGURE 3:**
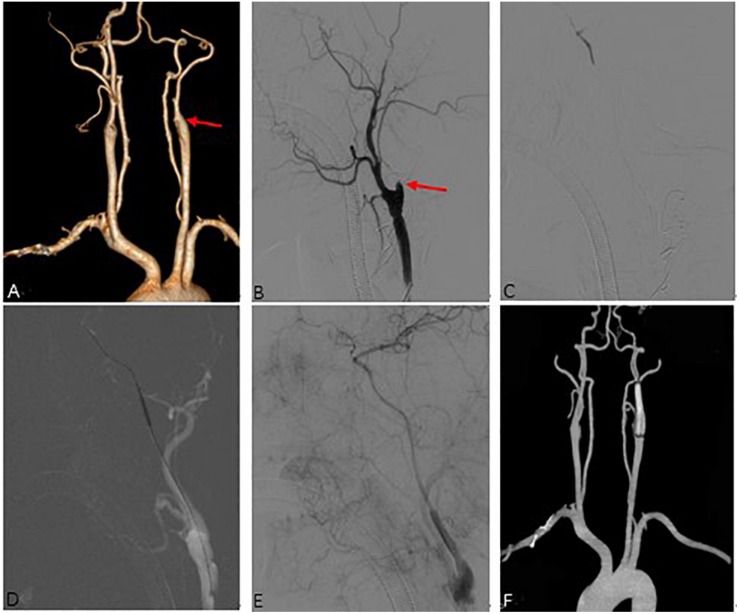
Representative Case 3. A 53-year-old man with right-sided limb weakness underwent hybrid surgery for internal carotid artery occlusion (ICAO). **(A)** Preoperative computed tomography angiography (CTA) showed left total ICAO (red arrow). Intraoperative angiography showed **(B)** an artificial stump (red arrow) after carotid endarterectomy and distal true lumen of ICA **(C)**. Successful recanalization **(E)** was achieved after balloon expansion **(D)** and deployment of the stent. **(F)** CTA showed a patent ICA at 3 months after the surgery.

The DSA and CTA images were assessed by two neurosurgeons. The occlusion length, occlusion site, collateral circulation, clinical variables, and neurologic data were collected and analyzed (Figures 1A, 2A, 3A). The occlusion length was measured from the occlusion proximal site to the distal reconstituted ICA on lateral angiographic image, ignoring potential curvature of the occluded segment. Brain perfusion MRI (MRP), CTP (Figures 1E–H) or PET was performed to evaluate the cerebral hemodynamics and perfusion–diffusion mismatch. Patients received medical therapy that consisted of dual antiplatelet therapy with aspirin (100 mg/day) and clopidogrel (75 mg/day) for at least 5 days before surgery.

### Hybrid Surgery Procedure

The recanalization procedure was performed in a hybrid operating room equipped with an angiographic fluoroscopy system. Under general anesthesia, all patients underwent standard CEA. A skin incision was made along the anterior border of the ipsilateral sternocleidomastoid muscle to explore the common carotid artery (CCA), ICA, and external carotid artery (ECA). An arteriotomy was made on the ICA bifurcation after clamping of the superior thyroid artery, CCA, ICA, and ECA. After removal of the atherosclerotic plaque (Figures 2B, 3B), the ICA clamp was loosened to observe arterial backflow. If little or no blood backflow was present, the surgical wound was sutured with placement of drainage; this was followed by EI therapy via percutaneous transfemoral approach.

A size 8 French artery sheath was positioned via transfemoral approach, and angiography was performed to observe the patency of ICA and formation of dissection. At the same time, intravenous infusion of heparin was carried out intraoperatively to maintain activated clotting time of 200–250 s. A 8F guiding catheter was placed into CCA or ICA, and a 0.014-inch Transend microwire (Stryker Neurovascular, Fremont, CA, United States) or Pilot microwire (Abbott Vascular, Santa Clara, CA, United States) in combination with Echelon-10 microcatheter (Medtronic, Minneapolis, MN, United States) or Gateway balloon catheter (Stryker Neurovascular, Fremont, CA, United States) were used to penetrate through the occluded segment of ICA (Figure 3C). After successfully reaching the distal true lumen of ICA, a Spider embolic protection device (ev3 Neurovascular, Irvine, CA, United States) was used if an adequate landing zone was identified, otherwise a Mo. Ma occlusion system (Medtronic, United States) was used to protect from embolization in cases with long or intracranial occlusion. Then a 2F or 3F Fogarty balloon catheter (Edwards Lifesciences Corp. Irvine, CA, United States) was sent into the distal true lumen to remove the thrombus, and this operation could be repeated two or three times. If no robust backflow of blood was present, a selective angiography was performed to evaluate the occlusion. Next, a Gateway balloon (Boston Scientific Corporation, Natick, MA, United States) dilation was performed distally (Figure 3D), along with sterling Monorail angioplasty balloon (Boston Scientific, United States) to reconstruct the proximal carotid artery lesion, followed by deployment of a Wallstent (Boston Scientific, United States) for dissection or stenosis of cervical ICA, or Winspan stent (Boston Scientific, United States) and Enterprise stent (Codman Neuro, New Brunswick, NJ, United States) for distal lesion. Post dilation with balloon was performed if remnant stenosis within the stent was over 50%. Wiring was stopped if the futile attempt was more than 30 min. The recanalization was considered a success (Figures 1C, 2C, 3E) if the residual diameter stenosis of occluded segment was less than 20%, and grade 3 antegrade flow was established by using Thrombolysis in Cerebral Infarction (TICI) Grade system (Almekhlafi et al., 2014), otherwise, the surgery was unsuccessful if no or little blood backflow was seen or endovascular equipment couldn’t cross the occlusion.

### Postoperative Management and Follow-Up

A postoperative CT scan was performed to identify intracranial hemorrhage or new ischemic lesions, and the blood pressure was usually controlled at <140/90 mmHg for 3 days. For patients without hypertension, the blood pressure was strictly controlled at <120 mmHg. Aspirin (100 mg/day), clopidogrel (75 mg/day), and atorvastatin calcium (20 mg/day) tablets were taken for at least 3 months in patients with stent placement, and a single antiplatelet agent and atorvastatin calcium were thereafter taken for life. CTA (Figures 1D, 2D, 3F), CTP (Figures 1I–L) or MRP was performed at 3 months after the operation, and DSA was performed at 6–12 months and annually afterward. Recurrent ischemic symptoms, reocclusion, the modified Rankin scale (MRS) score, the Barthel index score, and the Mini-Mental State Examination (MMSE) score were documented during follow-up.

### Statistical Analysis

The statistical analysis was performed using SPSS software 25.0 (IBM Corp., Armonk, NY, United States). Categorical variables are expressed as count (with percentage), and continuous variables are expressed as median or mean ± standard deviation. The paired *t* test (or *t* test) and χ^2^ test (or Fisher’s exact test) were used to compare continuous variables and categorical variables, respectively. Multivariate logistic regression was performed to identify the factors influencing the recanalization rate. A *P* value of <0.05 was considered statistically significant.

## Results

In total, 55 patients (10 women and 45 men) with a mean age of 59.3 ± 8.8 years were enrolled in the study. The patients’ demographic data are shown in Table 1. Of all 55 occluded ICAs, 43 were successfully recanalized with a success rate of 78.2%. All patients had a history of ischemic stroke or TIA. The median duration from diagnosis of occlusion to treatment was 100 days. The duration was significantly longer in the failure group than in the success group (median, 200 vs. 90 days, respectively; *P* < 0.01), but other variables between two groups were not statistically different (*P* > 0.05) (Table 1).

**TABLE 1:** Baseline characteristics of patients.

**Variables**	**Success group (*n* = 43)**	**Failure group (*n* = 12)**	**Total (*n* = 55)**	***P* value**
Age (years)	59.8 ± 7.7	57.4 ± 12.2	59.3 ± 8.8	0.532
Gender				0.430
Female	9 (20.9)	1 (8.3)	10 (18.2)	
Male	34 (79.1)	11 (91.7)	45 (81.8)	
Smoking	25 (58.1)	9 (75.0)	34 (61.8)	0.336
Drinking	8 (18.6)	2 (16.7)	10 (18.2)	1.000
Past history				
Hypertension	30 (69.8)	7 (58.3)	37 (67.3)	0.733
Diabetes	19 (44.2)	4 (33.3)	23 (41.8)	0.742
Hyperlipidemia	8 (18.6)	0 (0.0)	8 (14.5)	0.178
Coronary heart disease	2 (4.7)	0 (0.0)	2 (3.6)	1.000
Clinical presentation				0.7436
TIA	18 (41.9)	4 (33.3)	22 (40.0)	
Stroke	25 (58.1)	8 (66.7)	33 (60.0)	
Duration (days, median)	90	200	100	0.002

*Values are presented as mean ± standard deviation, median or *n* (%). TIA, transient ischemic attack.*

CEA was performed in all patients at first; balloon dilation was subsequently performed in 5 and stent implantation in 38, a total of 66 stents were used. The guidewire failed to pass through the distal occluded segment in the failure group. The lesion characteristics are summarized in Table 2. The occlusion length was significantly greater in the failure than success group (7.5 ± 1.0 vs. 5.4 ± 1.5 cm, respectively; *P* < 0.001). The success rates of distal ICA recanalization at the petrous segment or below, at the cavernous segment, and at the clinoid segment or above were 100, 33.3, and 14.3%, respectively (*P* < 0.001).

**TABLE 2:** Lesion characteristics and surgical types.

	**Success group (*n* = 43)**	**Failure group (*n* = 12)**	**Total (*n* = 55)**	***P* value**
Left lesions	22 (51.2)	6 (50)	28 (50.9)	1.000
Occlusion length (cm)	5.4 ± 1.5	7.5 ± 1.0	5.9 ± 1.7	<0.001
Level of distal ICA reconstitution				<0.001
Petrous segment or below	39 (90.7)	0 (0.0)	39 (70.9)	
Cavernous segment	3 (7.0)	6 (50.0)	9 (16.4)	
Clinoid segment or above	1 (2.3)	6 (50.0)	7 (12.7)	
Surgical types				
CEA + balloon dilation	5 (11.6)	0 (0.0)	5 (9.1)	
CEA + stent implantation	38 (88.4)	0 (0.0)	38 (69.1)	
CEA + EI (failed)	0	12 (100.0)	12 (21.8)	

*Values are presented as mean ± standard deviation, median or *n* (%). CEA, carotid endarterectomy; ICA, internal carotid artery. EI, endovascular intervention.*

The postoperative complications and outcomes are shown in Table 3. After successful recanalization, one patient developed hyperperfusion syndrome with minor subarachnoid hemorrhage. Another patient developed laryngeal nerve injury after CEA procedure in the failure group. Thus, the overall periprocedural complication rate was 3.6%. After proper treatment, both patients recovered completely. During follow-up (median, 10.7 months; range, 4–25 months), one patient (2.3%) developed ICA reocclusion in the success group, and two patients with unsuccessful recanalization experienced a minor ischemic stroke on days 97 and 152, respectively. CTA or perfusion CT was scheduled at 3 months and DSA at 6–12 months. The pre-procedural MRS score, MMSE score, and Barthel index score were similar between success group and failure group (Table 4). In the success group, 35 of 43 (81.4%) patients showed improvement in their symptoms, and their MRS score significantly improved (*P* < 0.001). Significantly different MRS score at 3 months follow-up was observed between groups (median, success group: 0 vs. failure group: 0.5, respectively; *P* < 0.05). No significant difference was found in the other scores (*P* > 0.05) (Tables 3, 4).

**TABLE 3:** Outcomes and postoperative complications.

	**Success group (*n* = 43)**			**Failure group (*n* = 12)**		
	**Baseline**	**3 months post-procedure**	**P value**	**Baseline**	**3 months post-procedure**	***P* value**
MMSE score	30	30	0.230	30	30	1.000
MRS score	1	0	<0.001	1	0.5	0.806
Barthel Index	100	100	0.874	100	100	1.000
Postoperative complications						
Hyperperfusion syndrome	1	0	–	0	0	–
Laryngeal nerve injury	0	0	–	1	0	–
Clinical outcome						
Ischemic events	0	0	–	0	2	–
Death	0	0	–	0	0	–
Reocclusion	0	1	–	–	–	–

*Values are presented as median or *n*. MMSE, Mini-Mental State Examination; MRS, modified Rankin scale. Ischemic events were defined as ischemic stroke, transient ischemic attack, or amaurosis fugax.*

**TABLE 4:** Neurocognitive and neurologic function among groups.

	**Success group (*n* = 43)**	**Failure group (*n* = 12)**	***P* value**
MMSE score			
Baseline	30	30	0.170
Post-procedure (3 months)	30	30	0.422
MRS score			
Baseline	1	1	0.901
Post-procedure (3 months)	0	0.5	0.036
Barthel index			
Baseline	100	100	0.694
Post-procedure (3 months)	100	100	0.748

*Values are presented as median.*

The duration, occlusion length, and level of distal ICA reconstitution with a *P* value of <0.05 were included in the multivariate logistic regression. The results demonstrated that the level of distal ICA reconstitution was the only independent factor affecting the success of hybrid surgery (*P* < 0.05), with an odds ratio of 2.517 and 95% confidence interval of 1.1 to 139.723 (Table 5).

**TABLE 5:** Factors affecting recanalization (multivariate analysis).

	**Odds ratio**	**95% CI**	***P* value**
Duration	0.012	0.993–1.032	0.207
Occlusion length	0.780	0.419–11.374	0.354
Level of distal ICA reconstitution	2.517	1.1–139.723	0.042

*CI, confidence interval; ICA, internal carotid artery.*

## Discussion

Chronic ICAO often leads to severe outcomes due to ischemic stroke in patients without sufficient collateral compensation. The OEF is a gold standard to assess the cerebral blood flow, If ipsilateral-to-contralateral OEF ratio is greater than 1.13 or symptomatic patients with stage I or II hemodynamic failure, surgical treatment is usually recommended (Powers et al., 2011; Kuroda et al., 2014). However, it is difficult to choose a viable surgical treatment. In recent years, hybrid surgery was proven to be a feasible and efficient treatment and increased the success rate of recanalization in patients with chronic long segment or tandem occlusion (Shih et al., 2013; Jiang et al., 2019; Li et al., 2019). The present study displayed the efficacy and safety of hybrid surgery and identified a predictor of successful recanalization.

The successful recanalization rate with a TICI score of 3 was 78.2%, which was higher than that of initial CEA or EI alone (Thompson et al., 1986; Chen et al., 2016). The baseline characteristics and lesion characteristics (except the duration, occlusion length, and level of distal ICA reconstitution) were statistically homogeneous between the two groups (Tables 1, 2). As some literature has reported, it is relatively straightforward to achieve recanalization for acute and subacute ICAO because the thrombus is soft and clot fibrosis does not occur (Namba et al., 2012). The clot develops fibrotic organization and extends gradually over time, which increases the difficulty of recanalization. In the present study, the longer duration of occlusion in the failure group (median, 200 days) supports the above-mentioned view, but recanalization could be achieved by using microwire with stiffer tip and catheter with a tapered tip, just as previous report (Jiang et al., 2019). However, the procedure must be performed carefully with above endovascular devices because the vessel wall can be easily perforated. Multiple projections, navigation of microguidewire in the center of vessel with good control, and low-pressure segmental angiography can reduce the risk of perforation and dissection of vessel wall. In theory, it’s difficult to negotiate the wire through the long ICAO due to the variable vessel course, and easily induces pseudoaneurysm formation and artery dissection, therefore, a longer occlusion of ICAO should reduce the technical success rate. The success group had a shorter occlusion lesion than the failure group, suggesting that a shorter lesion of ICAO increases the success of recanalization. In the present study, we classified occlusions by the level of distal ICA reconstitution (Figures 1B, 2A) and found that the success rates of recanalization at the petrous segment or below, at the cavernous segment, and at the clinoid segment or above were 100, 33.3, and 14.3%, respectively (*P* < 0.001). It seemed that recanalization rate of ICAO beyond petrous portion was lower, but Chen et al. (2016) reported that recanalization rate of ICAO with distal ICA reconstitution beyond clinoid segment was significantly lower. We speculated that our result was related to the small sample size and selection of patient group, larger studies should be necessary in the future.

Other factors, such as the stump morphology, atherosclerosis, and calcification in the initial part of the ICA or CCA, have been found to influence success rate of EI (Chen et al., 2016), The ICAO is often caused by atherosclerosis, with harder or severe calcified plaques that may hinder guidewire crossing and increase the difficulty for recanalization. However, the atherosclerosis plaque and dense calcium burden are mainly located at the proximal portion of the ICA because of the complex hemodynamics of low shear stress, flow stasis and flow separation at this site, not throughout the entire length of occlusion (Harrison and Marshall, 1977). CEA can remove the hard plaque at the initial part of the ICA or at the carotid bifurcation and build an artificial stump, then distal occlusion segment of ICA with loose calcification may be easily traversed, so factors above might have little effect on outcome in this study. The factors that may influence recanalization success were analyzed by multivariate analysis (Table 5). Our findings demonstrated that the level of distal ICA reconstitution was the only independent predictor of success of hybrid surgery for ICAO. This indicates that a lower location of the ICAO is associated with a higher success rate of recanalization. A future clinical study should consider stratified randomization for each level of ICAO.

In this study, the probability of any stroke during the postoperative period and follow-up was 2.3%, which is lower than that in some other reports (Powers et al., 2011; Jiang et al., 2019). Furthermore, no patient died and the complication rate was only 3.6%, indicating that a hybrid operation is a safe and efficient treatment. The incidence of complications is usually related to technical difficulties and uncertainties and excessive perfusion following recanalization. The patients’ systolic blood pressure was usually controlled at <140 mmHg after recanalization in our study, but it was strictly controlled at <120 mmHg if excessive perfusion was detected or hypertension was absent. Some reports have shown that CTP can be performed to assess the cerebral perfusion state and discover excessive perfusion early (Li et al., 2019), so a CTP was recommended after successful recanalization. A prominent technology-related complication is embolic events caused by distal migration of the thrombus during catheterization through the thrombus and balloon dilation or the deployment of the stent. Therefore, a distal embolic protection or proximal occlusion of CCA with occlusion balloon that could reverse blood flow was deployed to minimize the risk of embolic events in our study, so no embolic stroke or symptomatic stroke happened postoperatively. Among the patients with successful recanalization, the reocclusion rate at the 3-month follow-up was 2.3% (1/43), which is lower than that in some other studies (Lee et al., 2016; Jiang et al., 2019). However, the reason was presumably related to the shorter interval time after surgery. In those reports, balloon predilatation before stent placement and the occlusion site may have influenced the maintenance of the recanalization (Lee et al., 2016; Jiang et al., 2019).

In the present study, most patients (81.4%) showed improvement in their neurological symptoms and no patient had recurrent ischemia symptoms during 3-month follow-up after successful recanalization, but subjective improvement should not be used to evaluate the clinical success of the operation because of the natural development process of stroke. Previous studies have shown that successful treatment of ICAO may improve global cognitive function as well as attention and psychomotor processing speed (Lin et al., 2011; Fan et al., 2014). In those studies, a series of examinations and tests were performed to evaluate neurocognitive and neurologic function. With the exception of the MRS score, our findings were not statistically significant (Tables 3, 4), but the potential effectiveness of hybrid surgery cannot be ruled out. In summary, the neurological improvement, absence of recurrent TIA or stroke, and patency of the ICA on the basis of recanalization should be considered to indicate the potential effectiveness of the procedure. Further studies should give overall consideration to this issue and should include a longer follow-up period.

How to choose the optimal treatment for symptomatic ICAO remains controversial. Although CEA alone cannot achieve recanalization of long segmental occlusion or tandem occlusion, it can build an artificial stump, angle, and shorter lesion to enable better penetration with the endovascular equipment. With retrograde filling to the supraclinoid segment, endovascular therapy should be performed when the occlusion of the ICA has a stump or is in the acute and subacute stages. Hybrid surgery is more appropriate in chronic cases when long or tandem occlusion of the ICA has an atherosclerotic plaque or calcification and has no stump, furthermore, it does not require MCA occlusion and therefore has an advantage over EC-IC artery bypass. For occlusion of the ICA without a stump and supraclinoid filling, extracranial–intracranial artery bypass could be a therapeutic choice. In a previous study, Inoue et al. (2015) reported that surgical embolectomy might be considered as an additional therapeutic strategy to treat ICA terminus occlusion. It might also be useful to recanalize tandem occlusion at the initial part and the terminus of the ICA in conjunction with CEA. In another study involving multimodality *in situ* recanalization in a hybrid operating room (Jiang et al., 2019), EI was performed first for short occlusions or occlusions with a tapered residual root. Otherwise, CEA was chosen as the initial procedure. If the initial procedure failed, an alternative procedure was immediately performed. In that study, the hybrid procedure fully exhibited its advantage in terms of efficiency (Jiang et al., 2019). Therefore, it is critical to evaluate the occlusion before this operation.

### Limitations

This study had several limitations. First, it was a retrospective study, and some defects in the study design cannot be ignored (e.g., small sample size, single-center design with enrollment of only Chinese patients, and use of only one hybrid surgical method). In addition, the skill of the surgeons could lead to bias. Second, a longer follow-up is needed to evaluate neurologic improvement, the patency of the ICA, and the efficacy of hybrid surgery. Third, further exploration of treatment is required for patients with unsuccessful recanalization. Further studies are needed to take these issues into consideration and confirm the efficacy of hybrid surgery.

## Conclusion

Hybrid surgery might be a safe and efficient therapeutic method for symptomatic chronic ICAO, with favorable clinical outcomes and low periprocedural complications. The level of the distal ICA reconstitution may predict technical success. Therefore, it is prudent to recanalize the occlusion at the supraclinoid segment and with a long duration.

## Data Availability Statement

All datasets generated for this study are available on request.

## Ethics Statement

The studies involving human participants were reviewed and approved by the Institutional Ethics Committee of Beijing Tiantan Hospital. The patients/participants provided their written informed consent to participate in this study.

## Author Contributions

YY and JZ contributed the conception, design, and data analysis, and drafted the manuscript. YY and XL collected and analyzed the data. YY drafted and reviewed the manuscript. XL, RW, YZ, and DZ collected the data and revised the manuscript.

## Conflict of Interest

The authors declare that the research was conducted in the absence of any commercial or financial relationships that could be construed as a potential conflict of interest.
